# Retraction Note: Functional kinomics establishes a critical node of volume-sensitive cation-Cl^–^ cotransporter regulation in the mammalian brain

**DOI:** 10.1038/s41598-026-66445-w

**Published:** 2026-08-25

**Authors:** Jinwei Zhang, Geng Gao, Gulnaz Begum, Jinhua Wang, Arjun R. Khanna, Boris E. Shmukler, Gerrit M. Daubner, Paola de los Heros, Paul Davies, Joby Varghese, Mohammad Iqbal H. Bhuiyan, Jinjing Duan, Jin Zhang, Daniel Duran, Seth L. Alper, Dandan Sun, Stephen J. Elledge, Dario R. Alessi, Kristopher T. Kahle

**Affiliations:** 1 MRC Protein Phosphorylation and Ubiquitylation Unit, College of Life Sciences, University of Dundee, Dundee, DD1 5EH Scotland; 2 Departments of Neurosurgery and Pediatrics, Yale School of Medicine, New Haven, CT 06511 USA; 3 Division of Genetics, Brigham and Women’s Hospital, Boston, MA 02115 USA; 4 Department of Neurology, University of Pittsburgh School of Medicine, Pittsburgh, PA 15213 USA; 5 Department of Cancer Biology, Dana-Farber Cancer Institute, Boston, MA 02115 USA; 6 Department of Biological Chemistry & Molecular Pharmacology, Harvard Medical School, 250 Longwood Ave, SGM 628, Boston, MA 02115 USA; 7 Department of Neurosurgery, Massachusetts General Hospital, Boston, MA 02114 USA; 8 Division of Nephrology and Department of Medicine, Beth Israel Deaconess Medical Center, Boston, MA 022154 USA; 9 Department of Medicine, Harvard Medical School, Boston, MA 022154 USA; 10 Department of Cardiology, Howard Hughes Medical Institute, Boston Children’s Hospital, Boston, Massachusetts 02115 USA; 11 Veterans Affairs Pittsburgh Health Care System, Geriatric Research,Educational and Clinical Center, Pittsburgh, PA USA; 12 Department of Genetics, Harvard University Medical School, Howard Hughes Medical Institute, Boston, Massachusetts 02115 USA; 13 Departments of Pediatrics and Cellular & Molecular Physiology; Interdepartmental Neuroscience Program; and Centers for Mendelian Genomics, Yale School of Medicine, New Haven, CT 06511 USA; 14 Present Address: División de Investigación, Facultad de Medicina, UNAM, Mexico, México

Retraction of: *Scientific Reports* 10.1038/srep35986, published online 26 October 2016

The Editors have retracted this Article.

Following publication, concerns regarding Figure 3C were brought to the attention of the Editors. Six of the eight western blots presented in Figure 3C appear to be highly similar to an earlier publication of the authors^1^ where the experimental treatments described for the samples are different. The sample lanes of another blot are also fewer than expected. An institutional investigation by the University of Dundee did not find any laboratory records for the experiment Figure 3C is said to illustrate. The Editors therefore no longer have confidence in the reliability of this Article.

Jinwei Zhang, Gulnaz Begum, Mohammad Iqbal H. Bhuiyan, Seth L. Alper, Dandan Sun, Stephen J. Elledge, Dario R. Alessi & Kristopher T. Kahle agree with the retraction and its wording. Jinhua Wang, Arjun R. Khanna, Boris E. Shmukler, Paola de los Heros, Paul Davies, Jinjing Duan, Jin Zhang & Daniel Duran did not respond to correspondence about this retraction. The Editors were not able to obtain the current contact details for Geng Gao, Gerrit M. Daubner and Joby Varghese.
